# Supplementary material for: Splice-Junction-Based Mapping of Alternative Isoforms in the Human Proteome
Source: Cell Rep. Author manuscript; Available in PMC 2020 Jan 15. (PMC6961840; doi:10.1016/j.celrep.2019.11.026)
Supplement: 2 [file NIHMS1546469-supplement-2.zip › DF1/PXD010154/Testis/Testis_50_HDLBP_IGSCHHESFAEHR.pdf]

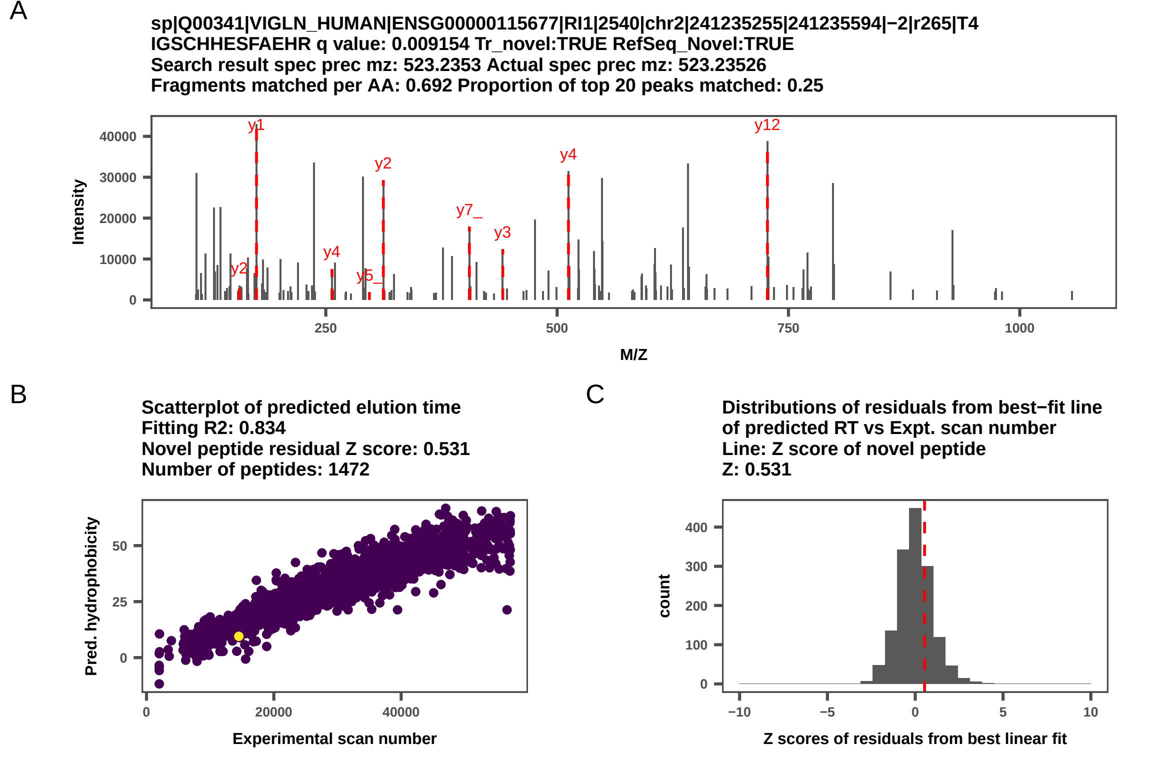
A
sp|Q00341|VIGLN_HUMAN|ENSG00000115677|RI1|2540|chr2|241235255|241235594|−2|r265|T4
IGSCHHESFAEHR q value: 0.009154 Tr_novel:TRUE RefSeq_Novel:TRUE
Search result spec prec mz: 523.2353 Actual spec prec mz: 523.23526
Fragments matched per AA: 0.692 Proportion of top 20 peaks matched: 0.25
Intensity
M/Z
y1
y2
y4
y5_
y2
y7_
y3
y4
y12
B
Scatterplot of predicted elution time
Fitting R2: 0.834
Novel peptide residual Z score: 0.531
Number of peptides: 1472
Pred. hydrophobicity
Experimental scan number
C
Distributions of residuals from best−fit line
of predicted RT vs Expt. scan number
Line: Z score of novel peptide
Z: 0.531
count
Z scores of residuals from best linear fit
